# Supplementary material for: PLGA-PEG-PLGA hydrogel with NEP1-40 promotes the functional recovery of brachial plexus root avulsion in adult rats
Source: PeerJ. 2021 Nov 1;9:e12269. doi: 10.7717/peerj.12269 (PMC8567856; doi:10.7717/peerj.12269)
Supplement: Supplemental Information 10 [file peerj-09-12269-s010.docx]

**Supplementary file**


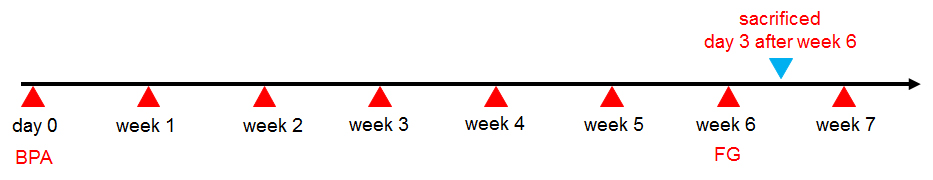


**Supplementary Figure 1:** The time point of fluorogold injection and sacrificed in rats. Three rats were randomly selected from each group at 6 weeks postoperatively. Then, these rats received FG injection and then received 3 days anti-infection treatment. Subsequently, the rats were subjected to cardiac perfusion using 4% paraformaldehyde.


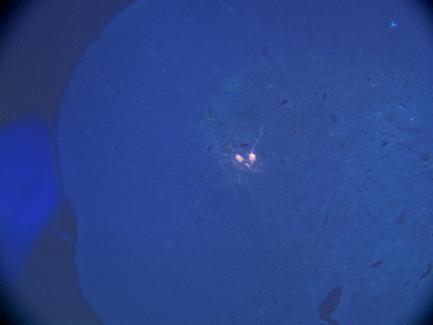


**Supplementary Figure 2**: The original picture of Figure 5 F in the manuscript, which shows the FG nerve retrograde labeling of anterior horn motoneurons of the C5-C8 segments in control group.


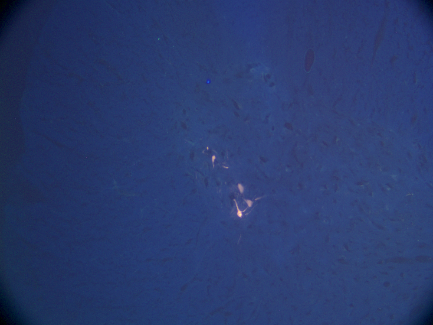


**Supplementary Figure 3**: The original picture of Figure 5 G in the manuscript, which shows the FG nerve retrograde labeling of anterior horn motoneurons of the C5-C8 segments in blank hydrogel group.


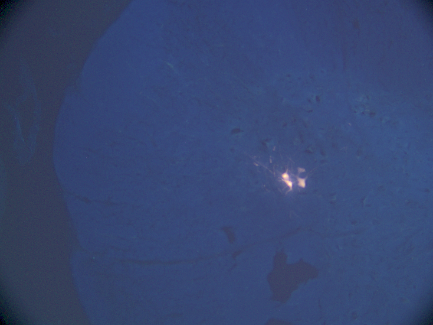


**Supplementary Figure 4**: The original picture of Figure 5 H in the manuscript, which shows the FG nerve retrograde labeling of anterior horn motoneurons of the C5-C8 segments in NEP1-40 group.


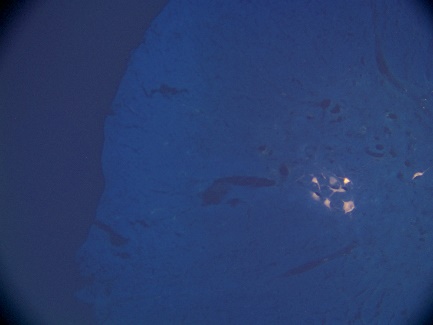


**Supplementary Figure 5:** The original picture of Figure 5 I in the manuscript, which shows the FG nerve retrograde labeling of anterior horn motoneurons of the C5-C8 segments in NEPl-40-loaded hydrogel group.
